# Detection of genomic deletions in rice using oligonucleotide microarrays

**DOI:** 10.1186/1471-2164-10-129

**Published:** 2009-03-25

**Authors:** Myron Bruce, Ann Hess, Jianfa Bai, Ramil Mauleon, M Genaleen Diaz, Nobuko Sugiyama, Alicia Bordeos, Guo-Liang Wang, Hei Leung, Jan E Leach

**Affiliations:** 1 Program in Plant Molecular Biology, Department of Bioagricultural Sciences and Pest Management, Colorado State University, Fort Collins, USA; 2 Department of Statistics, Colorado State University, Fort Collins, USA; 3 Gene Expression Facility, Kansas State University, Manhattan, USA; 4 Internation Rice Research Institute, Manila, Philippines; 5 Philippines, Institute of Biological Sciences, University of the Philippines, Los Baños, Philippines; 6 Department of Plant Pathology, The Ohio State University, Columbus, USA

## Abstract

**Background:**

The induction of genomic deletions by physical- or chemical- agents is an easy and inexpensive means to generate a genome-saturating collection of mutations. Different mutagens can be selected to ensure a mutant collection with a range of deletion sizes. This would allow identification of mutations in single genes or, alternatively, a deleted group of genes that might collectively govern a trait (e.g., quantitative trait loci, QTL). However, deletion mutants have not been widely used in functional genomics, because the mutated genes are not tagged and therefore, difficult to identify. Here, we present a microarray-based approach to identify deleted genomic regions in rice mutants selected from a large collection generated by gamma ray or fast neutron treatment. Our study focuses not only on the utility of this method for forward genetics, but also its potential as a reverse genetics tool through accumulation of hybridization data for a collection of deletion mutants harboring multiple genetic lesions.

**Results:**

We demonstrate that hybridization of labeled genomic DNA directly onto the Affymetrix Rice GeneChip^® ^allows rapid localization of deleted regions in rice mutants. Deletions ranged in size from one gene model to ~500 kb and were predicted on all 12 rice chromosomes. The utility of the technique as a tool in forward genetics was demonstrated in combination with an allelic series of mutants to rapidly narrow the genomic region, and eventually identify a candidate gene responsible for a lesion mimic phenotype. Finally, the positions of mutations in 14 mutants were aligned onto the rice pseudomolecules in a user-friendly genome browser to allow for rapid identification of untagged mutations .

**Conclusion:**

We demonstrate the utility of oligonucleotide arrays to discover deleted genes in rice. The density and distribution of deletions suggests the feasibility of a database saturated with deletions across the rice genome. This community resource can continue to grow with further hybridizations, allowing researchers to quickly identify mutants that harbor deletions in candidate genomic regions, for example, regions containing QTL of interest.

## Background

Mutants are critical tools for forward and reverse genetic approaches to dissect biochemical and metabolic pathways, and to determine gene function in plants. In the past few years, several strategies have been used to develop different rice mutant collections [1]. Although large collections of mutant lines were generated using T-DNA, *Ac*/*Ds*, and transposon insertions [1-3], they are limited to *japonica *rice varieties which are more amenable to transformation and regeneration than *indica *varieties. This is unfortunate, as *indica *varieties represent the predominant rice type grown in the world (~80%) and harbor many interesting traits important for rice production [4].

Genomic deletions induced by chemical and irradiation mutagens provide a rapid method to obtain a large mutant pool [5]. Advantages to these types of mutants are that they are relatively inexpensive to produce, any genotype can be used because there is no need for transformation, and the density of mutations generated allows for genome-wide saturation with relatively small populations. In rice, a collection of over 40,000 mutants induced by various chemical and irradiation strategies was developed in the *indica *rice cultivar IR64 [6]. IR64 was chosen because it is the most widely grown *indica *rice in Southeast Asia and because it contains a large number of valuable agronomic characteristics. The variety of mutagens was selected to ensure a collection with a range of deletion sizes, providing the opportunity to identify a mutation in a single gene or a deleted group of genes that might collectively govern a trait (e.g., quantitative trait loci, QTL). However, as the mutations in this collection are not tagged, time and labor intensive mapping strategies are needed to identify genes conferring interesting phenotypes. Alternative strategies for identifying untagged mutations have evolved in rice, with varying levels of technological difficulty and efficiency [7-12]. PCR-based strategies for reverse genetics use complex pools of mutant genomic DNA and PCR to detect deletions in genes of interest [7,8,11,12]. An example in rice is the 'deletagene' approach [8]. This approach requires an *a priori *hypothesis of what gene might be deleted. Further, it requires the design of flanking PCR primers that would amplify across a range of deletion sizes, because the size of the deletion and the number of genes in the deleted region would not be known. Targeting induced local lesions in genomes (TILLING) provides a reverse genetics technique to detect point mutations in genes of interest [9,10], but the detection and characterization of moderate to large deletions in rice remains tedious. None of these techniques are suitable for forward genetic screens.

With the completion of the rice genome sequencing projects and advances in microarray technology, comprehensive oligonucleotide microarrays are now available that can be used to discover genetic polymorphisms and deleted genes. Hybridization of genomic DNA to Affymetrix arrays has been used to discover single feature polymorphisms in Arabidopsis [13], rice [14], and barley [15]. Solid-support DNA arrays have been used for detection of deletions in the genome of Arabidopsis [16]. In addition, genomic DNA was hybridized to citrus spotted cDNA expression arrays to detect two hemizygous deletions induced by fast neutron in citrus [17]. Successful use of arrays for discovery of mutated genes is dependent on the proportion of the genome covered by the array, the size of the deletion (relative to the amount of coverage of an individual gene on the array), the complexity of the target genome. A key advantage of array hybridization is their potential for use in both forward and reverse genetics.

Our goal was to determine if oligonucleotide microarrays could be used to detect deletions mutations in rice, which has a genome size of 389 Mb [18], about three times the size of Arabidopsis. In a preliminary study, we used a proprietary custom Affymetrix oligonucleotide array [19] based on the Syngenta draft sequence of *Oryza sativa *ssp. *japonica *cv. Nipponbare [20], to show that hybridizing genomic DNA from mutants to oligonucleotide arrays could be used to identify known deleted regions in IR64, and therefore facilitate gene discovery (unpublished data). Although the chip was originally designed for use in expression-based experiments, the design was also ideal for genomic deletion detection because of the density of oligonucleotide probes for a given gene model (~11 probe pairs per gene model). The release of the Affymetrix Rice GeneChip^®^, which contains probe sets representing more than 50,000 transcripts  now provides a publicly available platform for hybridization-based deletion discovery.

In this study, we demonstrate the utility of the Affymetrix Rice GeneChip^® ^to discover deleted genes in rice. We describe a proof-of-concept experiment wherein we used hybridization intensity changes relative to wild type on a probe-by-probe basis to detect a known deletion on chromosome 5 in an IR64 mutant [6]. We demonstrate the utility of the technique as a tool in forward genetics in combination with an allelic series of mutants to rapidly narrow the genomic region and eventually identify a candidate gene responsible for a lesion mimic phenotype *spl1 *(spotted leaf 1). Finally, we align the positions of deletions in a total of 14 mutants onto the rice pseudomolecules in a user-friendly browser. The density and distribution of the deletions suggests the feasibility of creating a database describing a collection of available deletions in the genome. This community resource can continue to grow with further hybridizations, allowing researchers to quickly identify mutants that harbor deletions in candidate genomic regions containing QTL of interest. Previously reported array hybridization methods have focused on characterizing single feature polymorphism [13-15] or to identify deletions in forward genetics approaches [16,17,21]. We focus not only on the utility of this method for forward genetics, but also its potential as a reverse genetics tool through accumulation of hybridization data for a collection of deletion mutants harboring multiple genetic lesions.

## Results and Discussion

### Oligonucleotide microarray-based identification of deleted gene regions

The Affymetrix Rice GeneChip^® ^contains more than 55,000 probe sets representing 48,564 gene models based primarily on version two of the *japonica *cv. Nipponbare rice annotation provided by The Institute for Genomic Research (TIGR) and 1,260 *indica *transcripts . Although the oligonucleotides for the arrays were designed based primarily on *japonica *sequences and the IR64 mutant collection used in this study is an *indica *variety, all of our comparisons are based on changes in hybridization signals relative to the wild type IR64. Thus, differences in hybridization of *indica *rice DNA to *japonica *rice arrays are masked in the comparison.

**Figure 1:**
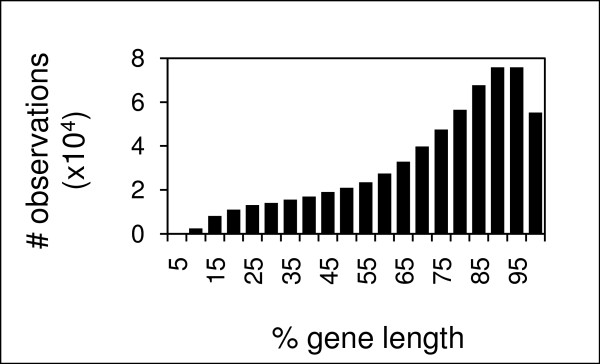
**Distribution of probes on the Affymetrix Rice GeneChip^® ^is biased to the 3' end of gene models**. Because absolute lengths of genes vary, the genes are represented as percentage of length. The Affymetrix probes were binned into 5% intervals along the gene length. The y axis represents the number of probes on the array within a bin.

To determine the efficiency of Rice GeneChip^® ^arrays to identify genomic deletions, we first analyzed the distribution of probes along the coding sequences. The data show a 3' bias in coding sequence representation (Figure 1). This is not unexpected as the array is designed to query expression data. However, promoters, introns and 5' genic regions are not or are less frequently queried in genomic DNA hybridizations as a result of the chip design, and deletions in these areas are thus less likely detected. Tiling arrays will likely provide better coverage of these regions.

### Array diagnostics and normalization

Prior to data analysis, the hybridization data was subjected to several diagnostic analyses. These include examination of variation in signal intensity, proportion of "present" calls, and any spatial anomalies (smudges, streaks, patches of extremely high or low signal) among the arrays [22]. Any arrays with a strong deviation from the wild type were discarded from the analysis. After passing diagnostics, a scale normalization of the data was performed. The log_2 _perfect match (PM) probe signals for each array were scaled such that the average for each array was the same as that for wild type. A benefit of this normalization method is that adding arrays to the analysis does not affect other arrays in the normalization scheme.

In preliminary studies, we tested the application of background correction to the data. However, while the background correction led to a higher power of detection, it also resulted in a higher false positive rate. This is because the background correction exaggerates probe level differences. While this is not a problem in "standard" microarray analysis, where probe values are summarized into a probe set summary statistic representing gene expression, it is a problem where probes are treated individually as in our analysis. For example, after background correction, roughly 3% of probes have a log_2 _ratio of -1 or less, but without background correction, less than 1% of probes have a log_2 _ratio of -1 or less. Thus, a background correction was not applied.

### Array hybridizations for detection of deleted regions

To reduce the costs associated with array-based deletion discovery, we explored the use of unreplicated hybridization data. The proposed analysis makes use of the multiple probes contained in each probe set. During the development phase, we performed replicate hybridizations of DNA from two different rice mutants (G650 and F1856), and determined that the use of a stringent log ratio [= log_2 _(mutant PM probe signal intensity/wild type PM probe signal intensity)] cutoff for a high proportion of probes within a probe set was almost equivalent to the use of a p-value cutoff. In addition, False Positive Rates (FPR1 and FPR2, based on two different methods of estimation described in Table 1) and True Positive Rates (TPR) were calculated to establish the parameters for calling deletions (Table 1). The FPR and TPR were determined from PCR-confirmation of deletions and non-deletions predicted from 14 array hybridization experiments. For example, using a log ratio cutoff ≤ -0.8 for at least 50% of probes in a probe set, we observed an FPR1 of 0, an FPR2 of < 0.0001, and a TPR of 0.767. While this information indicates that it is suitable to use a single array hybridization per mutant, replicates are recommended for the wild type line to ensure data consistency and allow for error rate examination based on wild type by wild type comparisons.

**Table 1:** True and false positive rates (TPR and FPR, respectively) for different log ratio [log_2_(mutant PM probe intensity/wild type PM probe intensity)] and (a) proportion (probes flagged/total probes in probe set).

**Log_2 _ratio**	**Proportion**	**TPR^b^**	**FPR1^c^**	**FPR2^d^**
-0.6	0.4	0.833	0.012	0.0015
-0.6	0.5	0.800	0	< 0.0001
-0.6	0.6	0.767	0	0
-0.6	0.7	0.600	0	0

-0.8	0.3	0.833	0	0.001
-0.8	0.4	0.833	0	0.0002
-0.8	0.5	0.767	0	< 0.0001
-0.8	0.6	0.633	0	0

-1	0.3	0.833	0	0.0002
-1	0.4	0.800	0	< 0.0001
-1	0.5	0.667	0	0
-1	0.6	0.600	0	0

^a^Analysis based on PCR confirmation 30 deletions and 82 non-deletions using primers described in Additional file 3.

^b^TPR was calculated as the proportion of PCR-confirmed deletions that are correctly called by the analysis.

^c^FPR1 is the proportion of PCR-confirmed non-deletions, that are correctly called deleted by the analysis.

^d^FPR2 is the proportion of probe sets meeting defined log ratio and proportion combinations for the wild type replicates, i.e., log_2_(WT_1_/WT_2_) and for log_2_(WT_2_/WT_1_).

The analysis used for deletion discovery allowed flexibility, depending on the end-user's tolerance for false positives or negatives. In this study, the specific parameters (log ratio and proportion) were selected using Table 1 as a guide. The log ratio [= log_2_(mutant probe signal intensity/wild type probe signal intensity)] for each probe was first determined and log ratios for flagging probes were selected at less than or equal to -0.6 or -0.8. Probe sets that had more than a defined proportion of probes (0.4–0.5), i.e., those with a log ratio ≤ -0.6 or -0.8, were called as potential gene model deletions.

**Figure 2:**
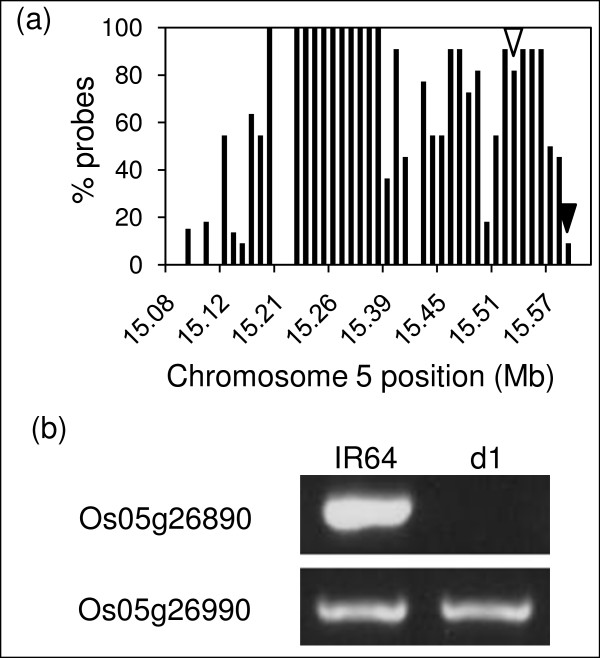
**Mutant line d1 contains a ~500 kb deletion on chromosome 5 encompassing the *RGA1 *gene**. a) Gene models in the region show a high percentage of probes with log_2_(mutant probe intensity/wild type probe intensity) ≤ -0.8, indicating a large deletion. b) PCR confirmation of the deletion of *RGA1 *(Os05g26890) relative to wild type (indicated by an open arrowhead in part a) and PCR confirmation of the right border of the deletion (Os05g26990) relative to wild type (indicated by a closed arrowhead in part a). The left border was not resolved.

As an example of the process for detecting deletions, we hybridized genomic DNA from a rice dwarf mutant *d1 *with a known deletion in the single copy *RGA1 *gene, previously shown to be responsible for the dwarf phenotype [23], to a single array. The mutation was induced by gamma radiation and confirmed using PCR and DNA blot analysis (data not shown). We predicted a deletion on chromosome 5 that contains the gene model Os05g26890, the *RGA1 *gene (Figure 2). Nine of eleven probes in the Os05g26890 probe set showed a log ratio ≤ -0.8, or a proportion of 0.82, identifying *RGA1 *as deleted.

In addition to the deletion of *RGA1*, 44 other gene models were predicted to be deleted in the *d1 *mutant line at log ratio ≤ -0.8 for 50% or more of probes. An aggregation analysis was used to automate identification of genomic regions with an overrepresentation of gene models predicted to be deleted, and the models were mapped to a genome browser . The analysis revealed a large deletion in the *d1 *line on chromosome 5 spanning 30 gene models including the *RGA1 *gene (Figure 2). One end of the *d1 *chromosome 5 deletion is predicted to fall between the two TIGR v5 loci Os05g26926 and Os05g27050. The other end of the deletion could not be reliably predicted because of the presence of multiple adjacent repetitive elements. A second large deletion was detected in the *d1 *mutant line on chromosome 2 (see browser).

**Figure 3:**
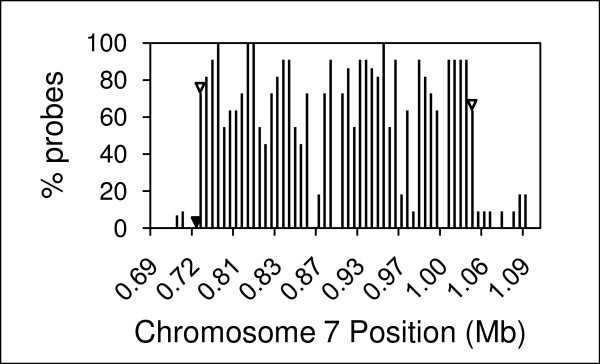
**Confirmation of a ~300 kb deletion on chromosome 7 in mutant line G282 as predicted by array hybridization using log ratio cutoff of < -0.8 for 50% or more of probes in a probe set**. Open arrowheads indicate deletions in gene models confirmed by PCR. The closed arrowhead indicates a gene model confirmed to be present by PCR.

In total, 14 rice mutants were screened using single array hybridizations and the putative deletions were mapped in a chromosome-by-chromosome display that shows the distribution of mutations across the 12 rice chromosomes . The browser allows for selection of different log ratio cutoffs, providing flexibility in data analysis. For example, in the total set of mutants, for probe sets with 50% or more probes showing a log ratio less than or equal to -0.6, the number of putatively deleted gene models ranged from 2 to 359 (Table 2). At this stringency, putative deletions were detected in all mutant lines, though some lines had many more than others. In mutant (G282), a high number of deletions were detected (Table 3). Increasing the stringency to log ratio -0.8 for 50% or more probes in a probe set revealed 89 deleted gene models in G282, with 43 deleted gene models on chromosome 7, suggesting a large deletion. The large deletion on chromosome 7 in G282 was also predicted by aggregation analysis to contain 46 gene models (Figure 3). This number is greater that the total number of deletions detected on chromosome 7 because not all probe sets within the putative deletion showed log ratio and proportions above the threshold. The deletion was confirmed by PCR. Large deletions detected in other mutants are shown in the browser.

**Table 2:** Number of deletions predicted on each chromosome in 14 individual IR64 mutants at log ratio < -0.6 for 50% or more probes in a probe set.

Chr	**Number of deleted gene models predicted per IR64 mutant line at log ratio < -0.6 for 50% or more probes in a probe set**														
	d1	D256	D2943	G282^a^	G650	G6458	G6489	G6603	G6686	G6728	G7534	G9799	F1856	F2045	Total

1	11	0	0	64	11	0	0	5	2	0	1	0	0	0	30
2	16	0	0	38	16	1	1	4	0	1	2	0	7	1	49
3	7	0	0	37	3	4	4	9	8	4	4	0	0	4	47
4	18	0	0	24	14	3	3	5	1	3	6	0	2	4	59
5	41	0	0	34	12	3	2	6	0	2	5	0	0	2	73
6	9	0	0	20	8	2	2	5	0	2	4	0	0	2	34
7	4	0	1	62	2	1	1	7	1	1	1	0	0	1	20
8	5	0	1	20	8	0	4	4	0	1	2	0	1	0	26
9	9	1	0	21	6	2	2	4	0	2	2	0	0	2	30
10	25	0	0	16	22	4	11	4	1	4	8	1	2	4	86
11	3	0	0	27	0	3	4	8	1	3	5	0	0	3	30
12	20	1	0	26	21	1	1	2	1	1	1	28	28	27	132

Total	168	2	2	359	123	24	35	63	15	24	41	29	40	50	616

^a^Deletions predicted for G282 are not included in totals. Because the number of predictions is large, to reduce FPR, a higher stringency (for example, log ratio < -0.8 for 50% or more probes in a probe set) is recommended for this mutant.

**Table 3:** Predicted probe set deletions using various combinations of log_2 _ratio and proportion (probes flagged/total probes) or adjacent probes including TPR and FPR rates as described in Table 1 and Additional file 2.

**Mutant line**	**Count of probe sets predicted to be deleted for different proportion (Prop) and log ratio (LR) combinations**				**Count of probe sets predicted to be deleted for different run length (RL)^a ^and log ratio (LR) combinations**		
	LR = -0.6 Prop = 0.5	LR = -0.8 Prop = 0.5	LR = -1.0 Prop = 0.3	LR = -1.0 Prop = 0.4	LR = -0.8 RL = 3	LR = -1 RL = 2	LR = -1 RL = 3

d1	168	45	50	39	63	66	39
D256	2	0	0	0	1	11	0
D2943	2	0	0	0	2	2	1
G282	359	89	139	69	333	560	109
G650	123	46	29	19	55	45	23
G6485	24	0	0	0	0	2	0
G6489	35	5	5	1	7	7	2
G6603	163	0	0	0	54	30	3
G6686	15	5	9	8	10	14	5
G6728	24	0	0	0	4	15	1
G7534	41	2	2	2	46	35	5
G9799	29	25	28	24	34	55	24
N1856	40	36	40	33	36	41	30
N2045	50	17	22	19	26	37	21
WT check	10	0	1	0	16	88	3

TPR	0.800	0.767	0.833	0.800	0.800	0.833	0.800
FPR1	0	0	0	0	0	0	0
FPR2	0.0002	< 0.0001	< 0.0001	< 0.0001	0.0008	0.004	0.0002

^a^Run length is group of adjacent probes within a probe set that meet a defined log ratio cutoff.

### Identification of overlapping mutated regions to target gene discovery

**Figure 4:**
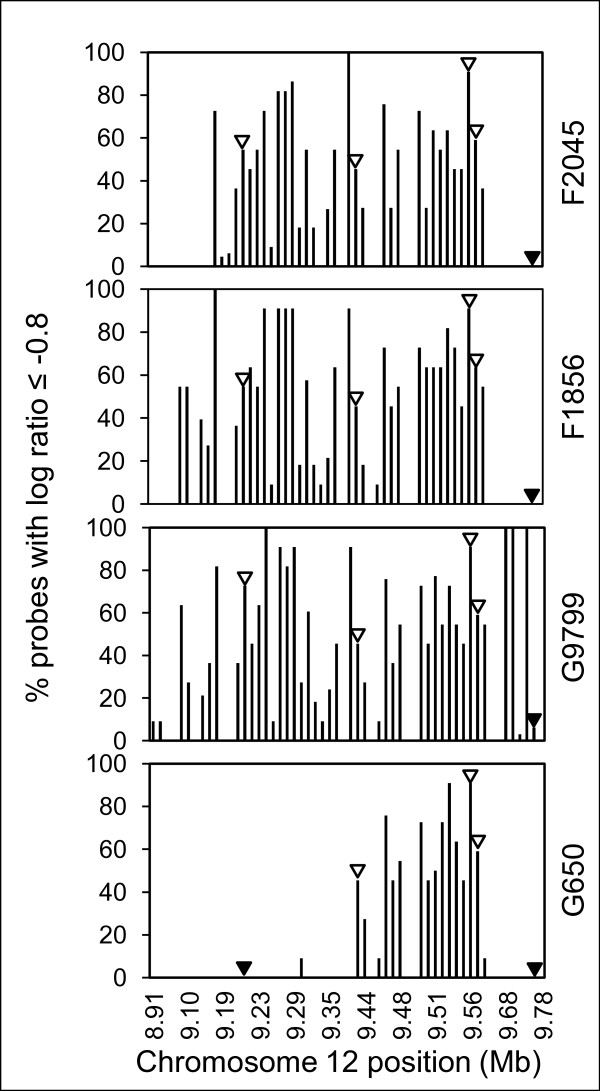
**Array-based deletion discovery identifies allelic relationships among *spl1 *mutants**. Hybridization of genomic DNA from two confirmed allelic *spl1 *mutants (G650 and F1856) and two mutants showing the distinctive *spl1 *lesion mimic phenotype (G9799 and F2045) identified overlapping deletions in all four lines on chromosome 12. A log ratio cutoff of ≤ -0.8 for 50% or more of probes in a probe set was used. Open arrowheads indicate deletions in gene models confirmed by PCR. Closed arrowheads indicate gene models confirmed to be present by PCR.

The rice mutant lines induced by chemical or irradiation strategies likely contained multiple deletions in the genome. We tested if hybridization of DNA from multiple mutant lines that exhibit a phenotype of interest could provide convergent data to identify the mutated region responsible for the phenotype and limit that region to the fewest gene models. Four mutants exhibiting the *spl1 *lesion mimic phenotype were selected as proof of concept. Two mutants had been genetically confirmed by complementation testing to be allelic at the *Spl1 *locus (G650 and F1856). Two additional mutants were included that displayed the *spl1 *phenotype, but had not been genetically confirmed (G9799 and F2045). For the four mutants, we predicted a total of 242 gene models to be deleted throughout the genome (Table 2). However, the four mutants showed overlapping deletions only on chromosome 12, and these deletions were located within the region where the *spl1 *mutation was previously mapped [24] (Figure 4). Selected candidate gene models predicted to be deleted in this region were validated by PCR. Thus, with a total of four hybridization experiments (one per mutant line), the location of the mutation conferring the phenotype was narrowed to a 70 kb region (21 gene models) on chromosome 12.

**Figure 5:**
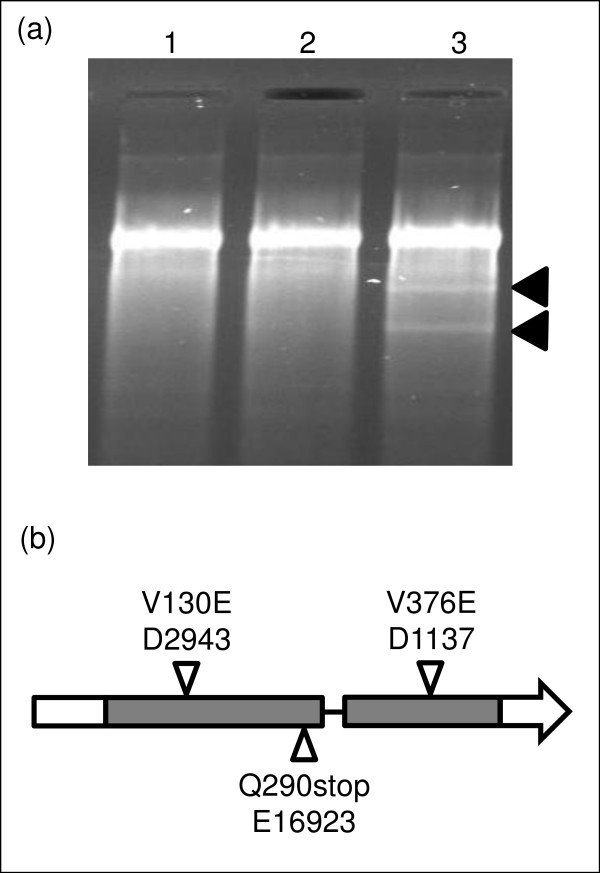
**Identification of a cytochrome P450 family member as a candidate for *Spl1***. Candidate genes located in the *Spl1 *region by array hybridization (Figure 4) were screened for SNPs in an EMS-generated mutant showing the *spl1 *phenotype by TILLING. (a) Detection of heteroduplex by TILLING between DNA for the rice mutant E16923 and wild type parent IR64 PCR products specific for LOC_Os12g16720 (a cytochrome P450 family member). Lanes 1 and 2 are CEL1 treatments of IR64 and E16923 amplicons, respectively. Lane 3 shows the activity of CEL1 enzyme on a heteroduplex generated between IR64 and E16923 amplicons. (b) Sequencing the amplified cytochrome P450 family member from E16923 confirmed the presence of a SNP at position 290 that resulted in a stop codon. Sequence data from two DEB mutants, D1137 and D2943, showing the *spl1 *phenotype revealed SNPs in LOC_Os12g16720 that caused amino acid changes.

Due to their small size, the diepoxybutane-derived (DEB) mutations were not reliably detected by array hybridization. However, they and ethylmethanesulfonate-derived (EMS) mutants were useful for confirming the location of the *spl1 *gene after delimiting the mutation to a few gene models by array hybridization. TILLING experiments using E16923 (shows *spl1 *phenotype) focused on gene models within the predicted deletion, and, after sequencing, revealed a point mutation resulting in an in-frame, premature stop codon in the first exon of Os12g16720, a member of the cytochrome P450 gene family (Figure 5). Sequencing of the entire gene from two DEB-derived mutants D1137 and D2943 (confirmed to be *spl1 *alleles by genetic complementation [25]) also showed single nucleotide polymorphisms (SNPs) in the Os12g16720 gene model. These SNPs were predicted to result in amino acid changes within the gene product that could cause the *spl1 *lesion mimic phenotype (Figure 5).

### Prediction of deletion sizes

We estimate that gamma ray and fast neutron produce both large (70 to 500 kb, Figures 2, 3, 5) and small deletions (see browser) within a single gene model. Sometimes, in what appeared to be large deletions, we observed apparently undeleted probe sets bracketed by deleted probe sets (e.g., in Figure 2 note the break in the deleted region in mutant *d1*). On closer inspection, several of these probe sets were found to be improperly mapped, gene family members or repetitive elements.

### Limitations

Though array hybridization and analysis proved a powerful tool for identifying deletions in rice, a limitation to the use of this method is the difficulty in detecting deletions in gene family members and other repetitive elements. An advantage of using mutagens that result in large deletions is the possibility of detecting mutations knocking out tandem-duplicated gene family members – a difficult mutation to obtain by traditional mutagenesis methods. Additionally, during mutagenesis, it is possible for large fragments of DNA to recombine in remote locations in the genome. Such a case has been demonstrated in the analysis of a gamma ray-induced mutation (G978), where a deletion event occurred on chromosome 12, followed by reintegration of part of the gene into a neighboring region of the same chromosome (N. Sugiyama, unpublished data). The hybridization technique reported here is not able to detect such rearrangements, as the genomic DNA is still physically present. Mapping strategies are better suited to detect these genomic rearrangements.

Smaller deletions were less reliably detected. Detection of small deletions is theoretically possible with reduced stringencies, but is limited by probe coverage of the gene models and deletion size. The Affymetrix Rice GeneChip^® ^design is limited by the coverage of probes for a gene model (usually 11 25-mers) and the distribution of those probes over a gene (Figure 1). Using DEB to induce mutations at the *rosy *locus in *Drosophila*, Reardon, *et al*. [26] found that 43% of the mutants were deletions ranging from 50 bp to 8 kb. DEB has also been reported to induce point mutations [27]; we observed point mutations after DEB mutagenesis of rice (Figure 5).

### Comparison with existing microarray detection methods

In other reports, expression data was used to detect genomic deletions [13]. We hybridized cDNA from the *spl1 *mutant G650 to the Agilent Rice 22 k Oligo expression array. This array represents approximately 22,000 rice genes with 60-mer oligonucleotides. Two genes, LOC_Os12g16540 and LOC_Os12g16720, were identified that were significantly down-regulated compared to wild type (see Additional file 1). These two genes were also detected as deleted by hybridization of the genomic DNA to the Affymetrix arrays (Figure 4). Indeed, LOC_Os12g16720 is the gene model that we identified as *Spl1 *(Figure 5). However, other genes shown to be deleted by hybridization of genomic DNA were not detected as deleted in the expression experiments. This is because relying on the absence of gene expression to detect a deletion assumes that the gene's expression would be detectable in wild type, which may not be the case. Using hybridization data from genomic DNA, all genes in wild type will be equally represented, regardless of mRNA expression level.

The experimental design reported here to detect deletions differs from previous studies, e.g., Gong et al [16], in several ways. First, our approach does not require development of advanced genetic populations. Second, because our goal was to develop a community resource, which maintains information on all deletions in genomes of each mutant, even those not contributing to a phenotype (see below), we did not use a pooling strategy to mask deletions unrelated to the phenotype. Preservation of information in a genome browser on all deletions in the same lines is important for researchers investigating the functions of other genes. Finally, we used a single hybridization per mutant to reliably detect deletions, and show the availability of an allelic series provides an advantage in quickly delimiting deleted regions responsible for a phenotype (Figure 4).

In addition to differences in the experimental design, the analysis reported here differs from other reports. Like Gong et al. [16], our calls are based on differences between individual perfect match probe intensities when comparing mutant to wild type arrays. They relied on adjacent probes, while we relied on the proportion of probes within a probe set and an aggregation analysis. For a fixed log ratio threshold, if a proportion and number of adjacent probes are chosen such that both methods have the same TPR, the FPR level is frequently higher when using the adjacent probes criteria (Table 1 and Additional file 2). The higher FPR may occur because many of the probe sets on the array contain probes that overlap often by more than 10 bases. In a case where these overlapping probes represent a region of variable hybridization efficiency, a few overlapping (adjacent) probes may produce low signal, while the rest of the probe set does not. In this case, relying on the "adjacent probe" method for deletion detection increases the FPR. Finally, to accurately detect larger deletions, we also used an aggregation analysis to delimit the potential borders of deleted regions.

### Feasibility of producing a deletion stock database for reverse genetics

Our long-term goal is to build a set of mutant lines with mapped deleted genomic regions that would serve the rice genetics community as a tool to study traits governed by multiple genes (QTL). Results from the analysis of the *d1 *and *spl1 *mutants demonstrate that deletions in multiple gene models are reliably detected by single array hybridizations. These non-target deletions detected in individual experiments, accumulated over time, can collectively provide a useful database for retrieving mutations in genes or regions of interest.

The data presented in this study suggest that it is feasible to develop a database of characterized mutants with deletions that span regions of interest in the genome. Assuming a median of 38 deleted gene models predicted at 80% TPR based on the 14 mutants analyzed (616 deletions/14 mutants, Table 2) and an coverage of ~38,000 gene models using Affymetrix Rice GeneChip^® ^(based on version 5 of the TIGR annotation, , there is a 91% probability of detecting a deletion in each gene model at least once using only 3,000 mutants. Currently, over 52,000 M_4 _mutant lines are maintained at IRRI; of these, approximately 15,000 are gamma ray-induced and 8,000 are fast-neutron-induced [4]. Thus, the available mutant collection is sufficient for near-saturation deletion mapping, provided that resources are available for analysis. Since a single array hybridization produces reliable data, the high costs usually associated with array experiments are minimized. Additionally, this collection will allow researchers to identify deleted regions that have been associated with QTL, presenting the possibility of using the collection to analyze the contribution of genes to complex phenotypes.

## Conclusion

This study demonstrates that deleted rice genes and genomic regions can be localized by hybridization of genomic DNA to oligonucleotide arrays. The approach is most reliable when used to detect mutations in single copy genes or large deletions, such as those produced by physical mutagens like gamma ray and fast neutron.

## Methods

### Mutants used in the study

A total of 14 mutants were used in this study; all were from the populations of mutants induced by treatment of the *indica *variety IR64 with DEB-treatment, FN or gamma ray (GR) exposure and were advanced to M_4 _or M_5 _lines prior to the experiment. Mutant *d1 *resulted from gamma ray mutagenesis and was confirmed to be deleted for the *RGA1 *gene by DNA blot analysis. The lesion mimic mutants, *spl1*, also known as *sl *(Sekiguchi lesion) [28], included six mutant lines, four of which had been confirmed by complementation tests to be allelic at the *spl1 *locus (D1137, D2943, G650 and F1856, DEB, GR and FN generated) and two genetically unconfirmed mutants (G9799 and F2045).

### Plant genomic DNA extraction and labeling

Genomic DNA was extracted from leaves of 45 day-old greenhouse-grown plants by CTAB extraction [29] and purified by cesium chloride gradient centrifugation [30]. The genomic DNA samples were assayed and quantified by spectrophotometry. Each sample was biotin labeled using the random priming method with BioPrime^® ^Array CGH Genomic Labeling System (Invitrogen, Carlsbad, CA) following the manufacturer's instruction. In brief, a total of 3 μg of genomic DNA from each sample was mixed with 40 μl of 2.5× random primer solutions. The final volume was adjusted to 88 μl with H_2_O. The reaction mix was denatured at 99°C for 5 min. Following the immediate cooling to 4°C, 10 μl of 10× dNTP mix containing biotin labeled dCTP and 2 μl of Exo Klenow fragments (80 units) were added to the reaction and incubated at 37°C for 2 h. Labeled DNA fragments were purified using the supplied column and assayed by gel electrophoresis prior to being applied to the arrays. Fragments of 100–200 bp were applied to the Affymetrix Rice GeneChip^® ^for hybridization.

### Target hybridization and image acquisition

Hybridizations were conducted according to Affymetrix standard protocol for eukaryotic target hybridization. Ten μg of biotinylated fragments were mixed in 200 μl with a final concentration of 0.1 mg/ml sonicated herring sperm DNA in a hybridization buffer with 100 mM 2-N-morpholino-ethane-sulphonic acid (MES), 1 M NaCl, 20 mM EDTA and 0.01% Tween 20, denatured at 99°C for 5 min and equilibrated at 45°C for 5 min prior to hybridization. The hybridization mix was then transferred into the Rice GeneChip^® ^cartridge and hybridized at 45°C for 16 h. The hybridized arrays were washed and stained using EukGE-WS2v5_450 protocol with an Affymetrix GeneChip Fluidics Station 450. The arrays were scanned twice and the intensities averaged with an Affymetrix GeneChip Scanner 3000 using GCOS 1.4.0.036 software (Affymetrix, Santa Clara, CA). The data discussed in this publication have been deposited in NCBI's Gene Expression Omnibus [31] and are accessible through GEO Series accession number GSE15071 .

### Data processing and analysis

Programming was done in R  and Bioconductor [32]. The "affy" package [33] was used to extract probe level information and examine diagnostics. In brief, the arrays were analyzed for spatial aberrations, congruence of signal distribution between arrays and variability in percentage of present calls across arrays [22]. Perfect match probe data was scale-normalized to the average of the wild type arrays. An R script was used to calculate log ratios versus wild type at the probe level. Probes meeting log ratio criteria (e.g., less than or equal to -0.8 log ratio on log_2 _scale) were flagged. Probe sets with more than 50% of probes meeting the defined log ratio criteria were called potentially deleted.

Analysis began with an initial combination of threshold (-0.8) and proportion (50%) values to generate a list of candidate deletions from individual arrays. Probe sets called deleted were aligned by BLAST [34] to the publicly available Nipponbare genome sequence [18] to identify location. For validation, sequence flanking the probe set location was used to design primers specific to the region. Genomic DNA from mutants and the wild type parent IR64 were used as template for PCR to confirm probe sets called deleted or not deleted on the arrays. PCR confirmation data from 112 amplifications was used to generate Table 1. TPR is calculated as the proportion of PCR-confirmed deletions that are correctly called by the analysis. FPR1 is calculated as the proportion of confirmed non-deletions incorrectly called deleted by the method. FPR2 is calculated by counting the number of probe sets meeting defined log ratio and proportion combinations for log_2_(WT_1_/WT_2_) and for log_2_(WT_2_/WT_1_), where wild type replicate 1 = WT1, and wild type replicate 2 = WT2. Primers used for validation are shown in Additional file 3.

### Aggregation analysis

Affymetrix rice probe sets were anchored to the Nipponbare genome using homology mapping of the probe sets to version 5 of the TIGR rice genome annotation gene models (data from ). The genome positions of the TIGR gene models were used for analysis in groups of genes along a chromosomal region. Probe sets mapped to multiple gene models were not included. The genome positions of the TIGR gene models were used for analysis in groups of genes along a chromosomal region. The ratios of deleted to non-deleted gene models within a predetermined genome block (0.5, 1.0 and 2.0 Mb) were compared to the genome-wide ratios using a Fisher exact test using a sliding window analysis with the window being shifted by one half block. Blocks significantly different from the fixed ratio (at p < 0.05) were declared as potentially contiguous deletions. Block size can be varied to determine deletion size with greater precision.

### Integration and visualization of deleted gene models and genomic regions using Generic Genome Browser

The coordinates of potential deletions in gene models and contiguous genome blocks were determined relative version 5 of the TIGR rice genome annotation . Probe set and deleted genome block information were coded using the General Feature Format (GFF version 2, or GFF2) and loaded directly into the genome visualization tool, Generic Genome Browser (GBROWSE) [35], which was preloaded with gene model annotation data. Each mutant with the corresponding GFF2 data was visualized against the rice genome as a separate track. Comparative visualization of the different mutants can be done by activating their respective track in GBROWSE.

### Tilling and sequence analysis

Tilling to identify SNPs was performed as described [36] using primers shown in Additional file 3. The putative gene conferring the *spl1 *phenotype, a cytochrome P450 family member, was amplified from mutants E16923, D1137, D2943, and wild type IR64 using gene specific primers (see Additional file 3). The amplicons were cloned into pGEM^®^-T Easy (Promega, Madison, WI), and the cloned PCR products were sequenced at the CSU Proteomics and Metabolomics Facility.

## Abbreviations

DEB: Diepoxybutane; FN: Fast neutron; GR: Gamma Ray; GBROWSE: GenericGenome Browser; (GFF): General Feature Format; (PM): Perfect match;(SNP): Single nucleotide polymorphism; (TILLING): Targeting induced local lesions in genomes.

## Competing interests

The authors declare that they have no competing interests.

## Authors' contributions

MB, JB, GLW, HL, and JEL conceived, designed, and supervised various aspects of the study. MB, JB, and MGD performed the DNA preparations and microarray processing, and MB carried out all PCR validations. AB performed genetic analysis of mutants and NS performed TILLING analyses. MB, AH, RM, HL and JEL contributed to the analysis and interpretation of the data. MB, HL and JEL drafted the manuscript. All authors read and approved the final manuscript.

## Supplementary Material

Additional file 1 **Volcano plot of expression data from rice *spl1 *mutant G650 shows significant down-regulation of genes which are candidates for deleted genes.** Data are from dual channel hybridizations comparing two rice lines, the *spl1 *mutant G650 and the wild type IR64. mRNA was extracted from the youngest fully expanded leaf of six plants each. cDNA was labeled with Cy3 and Cy5 dyes and hybridized onto the Agilent Rice 22 k Oligo Microarray. By plotting the log_2 _ratio of mutant/wild type signal intensity (x-axis) versus 1/log_10 _of the p-value (y-axis) for four array hybridizations, potential deletions were identified because they exhibited large negative fold changes coupled with significant p-values. Two such deleted genes are LOC_Os12g16540 and LOC_Os12g16720 (black circles). These genes were confirmed to be deleted by PCR and by hybridization of genomic DNA to the Affymetrix Rice GeneChip (Figure 4). Indeed, other methods, such as TILLING and sequencing, indicate that LOC_Os12g16720 is *Spl1*. However, other genes shown to be deleted by the hybridization of genomic DNA, such as LOC_Os12g16650 (Figure 4) do not have significant p-values or large negative fold changes from the expression profiles, indicating that these genes may not have been expressed in wild type plants during the time the tissue samples were taken. Click here for file

Additional file 2 **True and false positive rates (TPR and FPR, respectively) for different log ratio [log2(mutant PM probe intensity/wild type PM probe intensity)] and adjacent probe combinations.** True and false positive rates for the analysis method reported by Gong, et al. [16] Click here for file

Additional file 3 **Oligonucleotide primers used for validation of deletions and amplification of Spl1-gene candidates.** Table of primers used in the study Click here for file
