# Supplementary figures and images for: Detection of genomic deletions in rice using oligonucleotide microarrays
Source: BMC Genomics. 2009 Mar 25;10:129. doi: 10.1186/1471-2164-10-129 (PMC2666768; doi:10.1186/1471-2164-10-129)

## Slide 1
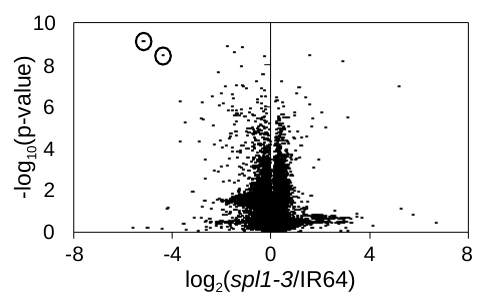

10
8
6
-log10(p-value)
4
2
0
-4
4
8
-8
0
log2(spl1-3/IR64)

Supplement: Additional file 1 — Volcano plot of expression data from rice spl1 mutant G650 shows significant down-regulation of genes which are candidates for deleted genes. Data are from dual channel hybridizations comparing two rice lines, the spl1 mutant G650 and the wild type IR64. mRNA was extracted from the youngest fully expanded leaf of six plants each. cDNA was labeled with Cy3 and Cy5 dyes and hybridized onto the Agilent Rice 22 k Oligo Microarray. By plotting the log2 ratio of mutant/wild type signal intensity (x-axis) versus 1/log10 of the p-value (y-axis) for four array hybridizations, potential deletions were identified because they exhibited large negative fold changes coupled with significant p-values. Two such deleted genes are LOC_Os12g16540 and LOC_Os12g16720 (black circles). These genes were confirmed to be deleted by PCR and by hybridization of genomic DNA to the Affymetrix Rice GeneChip (Figure 4). Indeed, other methods, such as TILLING and sequencing, indicate that LOC_Os12g16720 is Spl1. However, other genes shown to be deleted by the hybridization of genomic DNA, such as LOC_Os12g16650 (Figure 4) do not have significant p-values or large negative fold changes from the expression profiles, indicating that these genes may not have been expressed in wild type plants during the time the tissue samples were taken. [file 1471-2164-10-129-S1.ppt]
