# Supplementary material for: Detection of genomic deletions in rice using oligonucleotide microarrays
Source: BMC Genomics. 2009 Mar 25;10:129. doi: 10.1186/1471-2164-10-129 (PMC2666768; doi:10.1186/1471-2164-10-129)
Supplement: Additional file 2 — True and false positive rates (TPR and FPR, respectively) for different log ratio [log2(mutant PM probe intensity/wild type PM probe intensity)] and adjacent probe combinations. True and false positive rates for the analysis method reported by Gong, et al. [16] [file 1471-2164-10-129-S2.doc]

**Additional file 2.** True and false positive rates (TPR and FPR, respectively) for different log ratio [log_2_(mutant PM probe intensity/wild type PM probe intensity)] and adjacent probe combinations.

| **Log_2_ ratio** | **Adjacent probes** | **TPR^b^** | **FPR1^c^** | **FPR2^d^** |
| --- | --- | --- | --- | --- |
| -0.6 | 2 | 0.9 | 0.06 | 0.039 |
| -0.6 | 3 | 0.867 | 0.012 | 0.003 |
| -0.6 | 4 | 0.733 | 0 | <0.001 |
| -0.6 | 5 | 0.633 | 0 | 0 |
| -0.8 | 2 | 0.833 | 0 | 0.013 |
| -0.8 | 3 | 0.8 | 0 | <0.0001 |
| -0.8 | 4 | 0.5 | 0 | <0.0001 |
| -0.8 | 5 | 0.4 | 0 | 0 |
| -1 | 2 | 0.833 | 0 | 0.0004 |
| -1 | 3 | 0.8 | 0 | 0.0002 |
| -1 | 4 | 0.433 | 0 | 0 |
| -1 | 5 | 0.367 | 0 | 0 |

^a^Analysis based on PCR confirmation 30 deletions and 82 non-deletions using primers described in Table S2.

^b^TPR was calculated as the proportion of PCR-confirmed deletions that are correctly called by the analysis.

^c^FPR1 is the proportion of PCR-confirmed non-deletions, that are correctly called deleted by the analysis.

^d^FPR2 is the proportion of probe sets meeting defined log ratio and proportion combinations for the wild type replicates, i.e., log_2_(WT_1_/WT_2_) and for log_2_(WT_2_/WT_1_).
