# Supplementary material for: Detection of genomic deletions in rice using oligonucleotide microarrays
Source: BMC Genomics. 2009 Mar 25;10:129. doi: 10.1186/1471-2164-10-129 (PMC2666768; doi:10.1186/1471-2164-10-129)
Supplement: Additional file 3 — Oligonucleotide primers used for validation of deletions and amplification of Spl1-gene candidates. Table of primers used in the study [file 1471-2164-10-129-S3.doc]

Additional file 3. Oligonucleotide primers used for validation of deletions and amplification of Spl1-gene candidates.

| TIGR v5 locus^a^ | Primer^b^ | Sequence 5’ – 3’ | T_A_ (ºC)^c^ |
| --- | --- | --- | --- |
| LOC_Os07g02260 | EP1-1F | AGGAACGGCTGGAGGTAAGTAA | 57 |
|  | EP1-1R | ACACATTGTTGGACGCGTGA |  |
| LOC_Os07g02270 | HP1-1F | TCTCGATAGCTACATCCGGAAGTTG | 57 |
|  | HP1-1R | ACTTAAGATTTCGTATTGCCTCACACAT |  |
| LOC_Os07g02640 | CHP1-1F | GAGCACCTTGATTGAGCATGACTG | 57 |
|  | CHP1-1R | GTGCAAAGTTCCTGCGATCTCTTC |  |
| LOC_Os07g02800 | DBD1-1F | AGAAGAAGGGTGGTGATCGTAAGG | 54 |
|  | DBD1-1R | GGTACTTCTGCGCAATCAGAATCG |  |
| LOC_Os03g04490 | CDKI1-1F | GGGACCTCATCTCCCAATCGTTTA | 57 |
|  | CDKI1-1R | GTAGATTCCAGCCAGCATCCCTAT |  |
| LOC_Os04g47912 | EP2-1F | TAGCTCGATGTTCTCATCGTACCG | 57 |
|  | EP2-1R | CAGATGATGAAGTGGTATCTCCAGC |  |
| LOC_Os11g24170 | NBLRR1-1F | GCAGAACTAAAGGAGCAGTGCAAG | 57 |
|  | NBLRR1-1R | CACAGCCATGAAGATCGGTAATGC |  |
| LOC_Os08g25799 | MFTF1-1F | TGTGCCTTCAAGCTGTTTGGTC | 57 |
|  | MFTF1-1R | TGCCATCTCTTTGCCAGATTGC |  |
| LOC_Os08g26110 | DLP1-1F | GGTGTTCCAAACCGGAACGAAT | 57 |
|  | DLP1-1R | GCATTGGCAATTTGGCTTCGTG |  |
| LOC_Os08g26370 | HP4-1F | GAGGCAAGAGAGAGCATCAACA | 57 |
|  | HP4-1R | ATCGAAGGGTATAGTACCGCCA |  |
| LOC_Os04g21890 | RPM1-1F | GCAGGAACTACGATGGAAGAGCTA | 57 |
|  | RPM1-1R | CTTGGGAACCATCCCAGAACAAAC |  |
| LOC_Os02g06160 | LLPK1-1F | CAGTCAAGAAGTTTACACGTGCCG | 57 |
|  | LLPK1-1R | CAGATGATGAAGACCCTACTGGCA |  |
| LOC_Os05g26890 | GPA1-1F | TTGTATGATGTAGGAGGCCAGAGG | 57 |
|  | GPA1-1R | TTACAAGTTTCTGGTCTAGGGCCG |  |
| LOC_Os05g27050 | PTR2-1F | TATGCAAGTAGTGCAGGGTACGAG | 57 |
|  | PTR2-1R | CTGTTCCGGATTGTGTCCTGTTTG |  |
| LOC_Os06g09820 | UK1-1F | TGTACGCAAGGCTCTCTTACTAGC | 57 |
|  | UK1-1R | CTTCCATCTTCGGTGAAGGACTCA |  |
| LOC_Os10g34230 | CD5P-1F | TCAGGTCGTGGAAGTCATCTCAAG | 57 |
|  | CD5P-1R | CTGTAGCCATGTAGGTGTGGACTT |  |
| LOC_Os05g19360 | HP5-1F | CCTGTCGCCTAGCTCCTT | 59 |
|  | HP5-1R | AGGTCTCTCGGCAGTATTGTGTTC |  |
| LOC_Os02g27200 | MONOX1-1F | GTTTATAGGATGGCGCTTTCGGTG | 59 |
|  | MONOX1-1R | CTTAGCAAAGTCCCAACGGTGAAG |  |
| LOC_Os04g13140 | VP1-1F | AGAAACTAGCCGTAGCCTTGGTAG | 59 |
|  | VP1-1R | ATCATGGCGAGGCCACAGA |  |
| LOC_Os12g16690 | AK072042-2F | ATGCCGCAGCTAAATGTGGA | 56 |
|  | AK072042-1R | AACCTTGTGAGTGCCAACGA |  |
| LOC_Os12g16480 | Os1022121-4F | GATTGTTAGGAACCCAATCCCTCC | 56 |
|  | Os1022121-4R | CCTAGCAGAGACAACATGAACCTG |  |
| LOC_Os12g16720 | CytP450-4F | CTTGCTTCTCTGCTTAGTCACAATATT | 54 |
|  | CytP450-4R | CTTCCCCACTTAACCACGGTTAG |  |
| LOC_Os12g16720 | 3F | CCA ACA CAT CTC CAT TGC TG | 57 |
|  | cDR | CACTCGAAGTGGTAGAG |  |
| LOC_Os12g16720 | cDF | ATCATCAAGGAGACGTT | 57 |
|  | 4R | AACTGGCGCTACGCTACATC |  |
| LOC_Os12g16710 | Os1005492-4F | GTATGAAGGCCCATGTTACTACCG | 56 |
|  | Os1005492-4R | CATGGAGAGCTTGAGGAGGAGATA |  |
| LOC_Os12g16520 | Os1027066-4F | CTCCAGTAAATGACCACGGTTCTG | 54 |
|  | Os1027066-4R | GGGCAGTATGACTTGGAGAAGATG |  |
| LOC_Os12g16130 | Os1025420-1F | CTGTCACCATCAGAGACATCTTGG | 56 |
|  | Os1025420-1R | GGGATCAGGTCCACGAGAATTAAG |  |

^a^TIGR Rice Genome Annotation: <http://www.tigr.org/tdb/e2k1/osa1/>

**^b^**F = forward primer, R = reverse primer

^c^ T_A_ = annealing temperature
